# Combination versus sequential single agent chemotherapy in advanced breast cancer: associations with metastatic sites and long-term survival. The Western Cancer Study Group and The Southeastern Cancer Study Group.

**DOI:** 10.1038/bjc.1989.46

**Published:** 1989-02

**Authors:** R. T. Chlebowski, R. V. Smalley, J. M. Weiner, L. E. Irwin, A. A. Bartolucci, J. R. Bateman

**Affiliations:** Harbor-UCLA Medical Center, Division of Medical Oncology, Torrance 90509.

## Abstract

Two hundred and twenty-two patients with advanced breast cancer were randomised in two separate trials of similar design to either concomitant combination treatment or sequential use of the same drugs given as single agents changed only at disease progression. Both trials used cyclophosphamide, methotrexate, 5-fluorouracil and prednisone; the WCSG using triiodothyronine and the SECSG using vincristine as the remaining agent. A common data base was generated for these trials and combined for analysis. Considering all patients, combination treatment was associated with a significantly increased response (46 versus 25%, P less than 0.05) but not survival improvement. For the 141 patients without liver involvement, survival was closely comparable in both treatment arms. Combination therapy did result in significant survival benefit for patients with liver involvement (P less than 0.05). These studies demonstrate: (1) in the majority of breast cancer patients, sequential single agent therapy can result in survival comparable to combination treatment; and (2) sole consideration of response frequency does not represent the optimal criterion to compare therapeutic approaches in advanced breast cancer.


*Br. J. Cancer* (1989), **59**, 227–230 © The Macmillan Press Ltd., 1989

### Combination *versus* sequential single agent chemotherapy in advanced breast cancer: associations with metastatic sites and long-term survival

R.T. Chlebowski, R.V. Smalley, J.M. Weiner, L.E. Irwin, A.A. Bartolucci & J.R. Bateman

*The Western Cancer Study Group and the Southeastern Cancer Study Group*

**Summary** Two hundred and twenty-two patients with advanced breast cancer were randomised in two separate trials of similar design to either concomitant combination treatment or sequential use of the same drugs given as single agents changed only at disease progression. Both trials used cyclophosphamide, methotrexate, 5-fluorouracil and prednisone; the WCSG using triiodothyronine and the SECSG using vincristine as the remaining agent. A common data base was generated for these trials and combined for analysis. Considering all patients, combination treatment was associated with a significantly increased response (46 *versus* 25%, $P<0.05$) but not survival improvement. For the 141 patients without liver involvement, survival was closely comparable in both treatment arms. Combination therapy did result in significant survival benefit for patients with liver involvement ($P<0.05$). These studies demonstrate: (1) in the majority of breast cancer patients, sequential single agent therapy can result in survival comparable to combination treatment; and (2) sole consideration of response frequency does not represent the optimal criterion to compare therapeutic approaches in advanced breast cancer.

Metastatic adenocarcinoma of the breast has been treated with combination chemotherapy for over 20 years (Greenspan *et al.*, 1963; Cooper, 1969). During this period, many trials have reported higher response frequencies for combination chemotherapy compared to those achieved with single agent treatment (Henderson, 1987; Kieser & Conrad, 1987). Consequently, combination chemotherapy has become a standard approach for patients with metastatic breast cancer receiving chemotherapy (DeVita *et al.*, 1975). However, when parameters such as prolongation of survival and/or effective palliation of symptoms are used as criteria for effectiveness, greater difficulty in identifying a 'standard therapy' for metastatic breast cancer has become apparent (Hayes & Henderson, 1987). To this end, several recent breast cancer trial reports have emphasised long-term survival results in populations receiving therapeutic regimens with different intensity (Aisner *et al.*, 1987; Lopinzi & Ahmann, 1986; Taylor *et al.*, 1986).

In an attempt to define whether populations of advanced breast cancer patients can be successfully treated with regimens involving less intensive therapy, two large cooperative oncology groups, the Western Cancer Study Group (WCSG) and the Southeastern Cancer Study Group (SECSG) initiated similar chemotherapy trials for patients with metastatic breast cancer. These cooperative groups randomised patients between five drug combination chemotherapy and sequential use of single agent chemotherapy with the treatment agent changed when disease progression occurred. In both cooperative groups, higher response frequencies were observed on the combination treatment arm, and these trials were initially interpreted as supporting the superiority of combination over sequential therapy in advanced breast cancer (Smalley *et al.*, 1976; Chlebowski *et al.*, 1981). The long-term information on survival and response for subgroups of patients in these trials is the subject of the current report.

### Materials and methods

Between 1971 and 1973 the WCSG and SECSG entered patients on separate protocols comparing combination chemotherapy with five drugs to the sequential use of the same drugs given as single agents. Patient eligibility factors were similar in both studies and included: (1) histologically confirmed adenocarcinoma of the breast with metastases; (2) measurable disease parameters; and (3) no prior cytotoxic chemotherapy. Patients who either had failed hormonal therapy or had rapidly progressive disease were eligible for the WCSG study. In SECSG study, premenopausal patients were eligible only if they had prior ovarian oblation.

Pretreatment studies in both trials included: CBC, liver function tests, chest X-ray, liver scan and bone survey. Patients were also required to be less than 70 years of age, have an ECOG performance status 3 or greater, have a normal WBC (greater than 4,000 $\mu l^{-3}$), normal platelet count (greater or equal to 100,000 $\mu l^{-3}$) and normal hepatic function. After determination of eligibility, patients were randomly assigned to treatment arms using established procedures in their respective central statistical offices.

Identical response criteria were used in both trials. Responses were classified as: complete, complete disappearance of all measurable lesions with the appearance of no new lesions for a period greater than or equal to one month; partial, reduction of 50% of the cross-sectional area of all measurable lesions with the appearance of no new lesions for a period of greater or equal to one month; no change, minor change, or progression of any measurable lesion during therapy was considered as no response.

For this report, a common 48-item data base was abstracted from both SECSG and WCSG data sets and analysed at the WCSG Statistical Center. Curves representing survival were generated using the Kaplan–Meier method (Kaplan & Meier, 1958). Comparisons of survival between groups of patients was made with the Cox, Peto–Mantel test (Peto *et al.*, 1977). Patients categorised as having liver involvement were those in whom liver scan abnormalities consistent with metastatic disease were identified.

Both groups used the agents cyclophosphamide, methotrexate, 5-fluorouracil (5-FU) and prednisone in their trials. The WCSG used triiodothyronine and SECSG used vincristine as a fifth agent in the combination. Triiodothyronine was used in the WCSG trial since this agent has been reported to increase the response frequency in breast cancer patients treated with steroid (Lemon, 1957). Patients in the concurrent combination arms, referred to as combination, received all five agents during their initial course of therapy. The patients in the single agent sequential arms received the same drugs used in the combination arms, but given individually in sequence. Therapy with the initial single agent (5-FU in both trials) was continued until disease progression occurred, at which time the second single agent was begun. The details of the drug schedules used in both trials are outlined in Table I. The SECSG study used two schedules for their combination treatment. Chemotherapy given to

Correspondence: R.T. Chlebowski, Harbor-UCLA Medical Center, Division of Medical Oncology, 1000 W. Carson Street, Torrance, CA 90509, USA.
Received 9 May 1988, and in revised form, 22 September 1988.

**Table I** Schedule of chemotherapy

| | *WCSG* | *SECSG* | |
|---|---|---|---|
| Sequential single agent | 5-FU 15 mg kg$^{-1}$ week$^{-1}$ i.v. until relapse; then | 5-FU 600 mg m$^{-2}$ week$^{-1}$ i.v. until relapse; then | |
| | CTX 2 mg kg$^{-1}$ day$^{-1}$ p.o. until relapse; then | MTX 20 mg m$^{-2}$ biweekly p.o. until relapse; then | |
| | PRED 0.5 mg kg$^{-1}$ day$^{-1}$ p.o. and | CTX 100 mg m$^{-2}$ day$^{-1}$ p.o. until relapse; then | |
| | THY 0.005 mg day$^{-1}$ p.o. until relapse then | VCR 1 mg m$^{-2}$ week$^{-1}$ i.v. until relapse; then | |
| | MTX 30 mg m$^{-2}$ week$^{-1}$ i.v. | PRED 45 mg day$^{-1}$ for 14 day, 30 mg day$^{-1}$ for 14 days and 15 mg day$^{-1}$ for 30 days p.o. | |
| Concurrent combination | MTX 30 mg m$^{-2}$ biweekly i.v. beginning day 8 | MTX 30 mg m$^{-2}$ day$^{-1}$ 1, 8 p.o.; q28 days | MTX 20 mg m$^{-2}$ week$^{-1}$ p.o. |
| | 5-FU 15 mg kg$^{-1}$ biweekly i.v. beginning day 1 | 5-FU 400 mg m$^{-2}$ day$^{-1}$ 1, 8 i.v.; q28 days | 5-FU 400 mg m$^{-2}$ week$^{-1}$ i.v. |
| | THY 0.005 mg day$^{-1}$ p.o. | VCR 1 mg m$^{-2}$ day$^{-1}$ 1, 8 i.v.; q28 days | VCR 1 mg m$^{-2}$ week$^{-1}$ i.v. |
| | CTX 2 mg kg$^{-1}$ day$^{-1}$ p.o. | CTX 400 mg m$^{-2}$ day$^{-1}$ 1, i.v.; q28 days | CTX 100 mg day$^{-1}$ p.o. |
| | PRED 0.5 mg kg$^{-1}$ day$^{-1}$ p.o. | PRED 80 mg day$^{-1}$ 1–7; q28 days | PRED 45 mg day$^{-1}$ for 14 days, 30 mg day$^{-1}$ for 14 days p.o. and 15 mg day$^{-1}$ for 28 days |

patients after removal from study was similar in both arms of the WCSG trial with comparable numbers of patients in each arm receiving doxorubicin.

### Results

One hundred and twenty-six patients were randomised on the WCSG study with 121 of these evaluable for toxicity and response. One hundred and eleven patients were randomised in the SECSG trial with 101 evaluable for toxicity and reponse. The reasons for exclusion included concurrent hormonal therapy (six cases), gross protocol violations during early treatment (seven cases) and prior cytotoxic therapy (two cases). The survival curves (Figures 1 and 2) illustrate results from evaluable patients. Survival analyses were conducted including all entered patients with no change in any of the presented results. The patients receiving combination or single agent sequential treatment were comparable in both group trials in regard to age, disease-free interval and sites of metastatic involvement (Table II).

At this time, 210 of the 222 entered patients have expired. Patients have been followed for as long as 143 months after

**Figure 1** Life-table analysis of survival for all patients treated with combination as compared to those receiving single agent sequential chemotherapy in the WCSG and SECSG cooperative groups. n.s. indicates no significant difference.

**Figure 2** Life-table analysis of survival for patient subgroups treated with combination compared to those receiving single agent sequential therapy in the WCSG or SECSG cooperative groups. (**a**) Results in patients without liver metastasis (64% of all patients); (**b**) results in patients without liver or lung metastasis (30% of all patients); (**c**) results in patients with liver metastasis (36% of all patients).

**Table II** Pretreatment characteristics of patient groups

| | *Sites of metastases* | | | | *Performance score (median)* | *Age (median)* | *Free interval (<1 year)* | *% Premenopausal* |
|---|---|---|---|---|---|---|---|---|
| | *Lung* | *Liver* | *CNS* | *Bone* | | | | |
| Comb. (WCSG) | 49% | 49% | 11% | 57% | 74 | 56 | 44% | 32% |
| Seq. (WCSG) | 48% | 37% | 10% | 64% | 68 | 54 | 41% | 26% |
| Comb. (SECSG) | 40% | 28% | 9% | 56% | 73 | 55 | 32% | 40% |
| Seq. (SECSG) | 61% | 28% | 3% | 52% | 70 | 55 | 44% | 22% |

initiation of chemotherapy. The survival of all patients in the WCSG and SECSG trials is illustrated in Figure 1. As seen, there is no difference in survival for patients receiving combination as compared to sequential, single agent chemotherapy. Sixty-four per cent of patients in these trials were free of liver involvement as determined by a normal liver–spleen scan when treatment was begun. For this large subset of patients without liver metastases, survival on the sequential regimen was similar at all time periods to that seen with combination treatment (Figure 2). Thirty per cent of patients were free of both liver and lung involvement when treatment was begun. Only 6% of this group died within 8 months of entry regardless of treatment received. Survival for these patients without liver and lung metastasis was greater at all time periods with the sequential, single agent approach to therapy (median survival 18.4 months for single agent versus 14.3 months for combination chemotherapy, not significant). Thirty-six per cent of patients had evidence of liver metastasis when therapy was begun. For this group of patients with liver involvement, significant benefit (Figure 2) was associated with combination compared to single agent sequential treatment with prolongation of survival seen (median survival 10.1 months *versus* 5.3 months, respectively, $P<0.05$).

In both the WCSG and SECSG trials, objective responses and complete responses were seen more than twice as often in patients receiving the concurrent combination schedule (Table III). Duration of response was also significantly greater for the combination schedule in both the WCSG (median 13.4 months *versus* 7.7 months, $P<0.01$) and SECSG (median 9.2 months *versus* 5.1 months, $P<0.05$). Patients demonstrating at least a partial objective response lived longer than non-responding patients on both combination and sequential treatment ($P<0.001$) in both WCSG and SECSG trials.

Toxic effects of treatment are given in Table IV. Haematological toxicity was more common in patients from the WCSG trial. In both group trials, granulocytopaenia and thrombocytopaenia were somewhat more severe in patients receiving combination treatment. Deaths identified as being treatment-related occurred more commonly in patients on the combination arm (treatment related deaths occurring in 6% of combination *versus* 2% of sequential patients). A similar degree of gastrointestinal toxicity was noted with both approaches.

**Table III** Response frequency

| | *Total objective response* | *Complete response* |
|---|---|---|
| Comb. (WCSG) | 56% | 15% |
| Seq. (WCSG) | 26% | 3% |
| Comb. (SECSG) | 38% | 12% |
| Seq. (SECSG) | 18% | 6% |

**Table IV** Toxic effects of treatment regimens

| | *Treatment regimen* | | | |
|---|---|---|---|---|
| | *Combination* | | *Sequential* | |
| | *WCSG* | *SECSG* | *WCSG* | *SECSG* |
| Granulocytopaenia | | | | |
| <1,500 | 37% | 32% | 19% | 0% |
| <750 | 11% | 16% | 3% | 0% |
| Thrombopaenia | | | | |
| <100,000 | 11% | 16% | 8% | 9% |
| <50,000 | 8% | 6% | 3% | 6% |
| Treatment-related deaths | 3% | 9% | 0% | 6% |

### Discussion

In the present report, results from two separate chemotherapy trials were combined. These trials of the WCSG and SECSG were initiated at the same time and involved similar patient eligibility, treatment programmes and response characteristics. In addition, survival patterns in all examined subgroups were comparable in both trials. The similarity of study design, size and patient prognostic characteristics, and the relative homogeneity of results achieved in the WCSG and SECSG studies meet published criteria (Elashoff, 1978) for direct pooling of clinical trial results. Obviously, such criteria are much more stringent than those routinely employed in 'meta-analysis', which involves statistical comparison of outcomes from separate randomised trials (Sacks *et al.*, 1987; Dersimonian & Laird, 1986). If such stringent criteria can be met, the direct pooling of study populations provides a means of obtaining additional information from completed breast cancer trials.

Based on the increased response frequency and duration of response observed in both the WCSG and SECSG trials, these studies have been interpreted as supporting the superiority of combination chemotherapy over sequential, single agent treatment in breast cancer (Smalley *et al.*, 1976; Chlebowski *et al.*, 1981). This conclusion is certainly valid for breast cancer patients with liver involvement, who lived significantly longer when given initial combination chemotherapy. However, for the majority of patients with advanced breast cancer (those free of liver metastases), survival was at least as long on sequential treatment as on the combination regimen. The results from these trials suggest that ongoing combination chemotherapy may not be beneficial for all breast cancer patients.

In the current study, chemotherapy-related deaths were recognised three times more frequently with combination chemotherapy. Thus, the long-term survival of initially responding patients with disease in less threatening sites could have been adversely affected by unrecognised toxicity resulting from ongoing combination chemotherapy. Recently, increased thrombotic events were associated with combination chemotherapy in an adjuvant breast cancer therapy experience (Levin *et al.*, 1988). Recognition of other chemotherapy-related adverse effects may be more difficult in the advanced disease setting where patient symptoms are commonly associated with breast cancer progression.

Single agent chemotherapy has been compared to 13 combination chemotherapy regimens in randomised trials of patients with advanced breast cancer (Ahman *et al.*, 1974, 1987; Baker *et al.*, 1974; Canellos *et al.*, 1976; Chlebowski *et al.*, 1979; Hoogstraten *et al.*, 1976; Lemkin & Dollinger, 1973; Mouridsen *et al.*, 1976; Nemoto *et al.*, 1978; Rubens *et al.*, 1975; Smalley *et al.*, 1976). Despite the fact that combination chemotherapy resulted in significantly higher objective response frequency and complete response frequency in six of these trials with duration of response significantly higher in seven of these trials, no increase in overall survival was associated with combination compared to single agent chemotherapy in any trial. In one trial comparing CMF to melphalan, survival following the first year was greater for combination treatment, but overall survival benefit was seen in that study only in patients with liver metastases (Canellos *et al.*, 1976).

One prior study has compared sequential single agent chemotherapy beginning with 5-FU to the same agents given in concurrent combination (Baker *et al.*, 1974). In that trial, objective response frequency was somewhat higher for the sequential single agent regimen (53% versus 43%, not significant) and median survival was comparable (10.2 months for sequential *versus* 8.6 months for combination, not significant). One prior study compared 5-FU alone to four-drug combination chemotherapy and also reported similar response frequency on both arms (Lemkin & Dollinger, 1973). Three trials have compared doxorubicin alone to combination chemotherapy, but in no study has a significant overall survival difference emerged (Ahmann *et al.*, 1974; Hoogstraten *et al.*, 1976; Nemoto *et al.*, 1978). Thus, the published literature is in complete agreement with the conclusions generated by our current analysis. This information raises the testable hypothesis that less toxic treatment regimens may result in enhanced quality of life without detracting from overall survival for the majority of patients with advanced breast cancer.

The identification of a breast cancer patient population at low risk of early death regardless of initial treatment suggests that such patients could responsibly be given first line investigational treatment rather than 'standard' combination chemotherapy or hormonal therapy. This approach would facilitate evaluation of new approaches to advanced breast cancer management. In fact, the Cancer and Leukemia Group B (CALGB) has recently proposed use of first line investigational treatment for patients with advanced breast cancer (Hayes & Henderson, 1987). Consideration of pretreatment patient prognostic characteristics may facilitate the wider use of this approach in an easily defensible study design.

It is clear from these trials that combination chemotherapy is beneficial to breast cancer patients with liver involvement. These prospective results are in close agreement with conclusions retrospectively generated in a breast cancer population with liver metastases treated with single agent chemotherapy or combination chemotherapy at M.D. Anderson Hospital. In that report, median survival was 14 months for the group receiving combination chemotherapy compared to only five months for the patients receiving single agent chemotherapy (Zinser *et al.*, 1987).

In summary, sequential single agent chemotherapy results in survival comparable to combination chemotherapy for the majority of patients with metastatic breast cancer (those free of liver involvement). These data support the emerging interest in the use of overall survival and measures of disease palliation to compare effectiveness of different therapeutic approaches to advanced breast cancer management.

This study was supported by: WCSG, grants 3R10 CA05186-15 and CA08099-12; SECSG, grants CA07961-14 and CA24456-01.

### References

AHMANN, D., BISEL, H., EAGAN, R. & 3 others (1974). Controlled evaluation of Adriamycin (NSC-123127) in patients with disseminated breast cancer. *Cancer Chemother. Rep.*, **58**, 877.

AHMANN, D.L., SCHAID, D.J., BISEL, H. & 4 others (1987). The effect on survival of initial chemotherapy in advanced breast cancer: polychemotherapy versus single drug. *J. Clin. Oncol.*, **5**, 1928.

AISNER, J., WEINBERG, M., PERLOFF, R. & 6 others (1987). Chemotherapy versus chemoimmunotherapy (CAF *v* CAFVP *v* CMF each ± MER) for metastatic carcinoma of the breast: a CALGB study. *J. Clin. Oncol.*, **5**, 1523.

AUSTRALIAN AND NEW ZEALAND BREAST CANCER TRIALS GROUP (1986). A randomized trial in post-menopausal patients with advanced breast cancer comparing endocrine and cytotoxic therapy given sequentially or in combination. *J. Clin. Oncol.*, **4**, 186.

BAKER, L.H., VAUGHAN, C.B., AL-SARRAF, M. & 5 others (1974). Evaluation of combination versus sequential cytotoxic chemotherapy in the treatment of advanced breast cancer. *Cancer*, **33**, 513.

CANELLOS, G.P., POCOCK, S.J., TAYLOR, S.G. & 5 others (1976). Combination chemotherapy for metastatic breast carcinoma: prospective comparison of multiple drug therapy with L-Phenylalanine mustard. *Cancer*, **38**, 1982.

CHLEBOWSKI, R.T., IRWIN, L.E., PUGH, R.P. & 5 others (1979). Survival of patients with metastatic breast cancer treated with either combination or sequential chemotherapy. *Cancer Res.*, **39**, 4503.

CHLEBOWSKI, R.T., WEINER, J.M., RYDEN, V. & 4 others (1981). Factors influencing the interim interpretation of a breast cancer trial. *Controlled Clin. Trials*, **2**, 123.

COOPER, R.H. (1969). Combination chemotherapy in hormone resistant cancer. *Proc. Am. Assoc. Cancer Res.*, **10**, 15.

DERSIMONIAN, R. & LAIRD, L. (1986). Meta-analysis in clinical trials. *Controlled Clin. Trials*, **7**, 177.

DEVITA, V.T., YOUNG, R.C. & CANELLOS, G.P. (1975). Combination vs. single agent chemotherapy: a review of the basis for selection of drug treatment of cancer. *Cancer*, **36**, 99.

ELASHOFF, J.D. (1978). Combining results of clinical trials. *Gastroenterology*, **75**, 1170.

GREENSPAN, E.M., FIEBERM, M., LESTRICK, G. & 4 others (1963). Response of advanced breast carcinoma of the combination of the anti-metabolite methotrexate and the alkylating agent, thio-TEPA. *J. Mt Sinai Hospital*, **30**, 246.

HAYES, D.F. & HENDERSON, I.C. (1987). CAF in metastatic breast cancer: standard therapy or another effective regimen? *J. Clin. Oncol.*, **5**, 1497.

HENDERSON, I.C. (1987). Chemotherapy for advanced disease. In *Breast Diseases*, Harris, J.R., Hellman, S., Henderson, I.C. & Kinne, D.W. (eds) p. 428. J.B. Lippincott: Philadelphia.

HOOGSTRATEN, B., GEORGE, S.L., SAMAL, B. & 4 others (1976). Combination chemotherapy and adriamycin in patients with advanced breast cancer. *Cancer*, **38**, 13.

KAPLAN, E.L. & MEIER, P. (1958). Nonparametric estimation from incomplete observations. *J. Am. Stat. Assoc.*, **53**, 457.

KIESER, R. & CONRAD, B. (1987). Randomized clinical trials in breast cancer: a tabular summary. Part 2: advanced breast cancer. *Arch. Geschwulstforsch*, **57**, 323.

LEMKIN, S.R. & DOLLINGER, M.R. (1973). Combination vs. single drug therapy in advanced breast cancer. *Proc. Am. Assoc. Cancer Res.*, **14**, 37.

LEMON, H.M. (1957). Cortisone-thyroid therapy of metastatic mammary cancer. *Ann. Intern. Med.*, **46**, 457.

LEVINE, M.N., GENT, M., HIRSH, J. & 4 others (1988). The thrombogenic effect of anticancer drug therapy in women with stage II breast cancer. *N. Engl. J. Med.*, **318**, 404.

LOPINZI, C.L. & AHMANN, D.L. (1986). Chemotherapy versus hormonal therapy in advanced breast carcinoma. *N. Engl. J. Med.*, **315**, 1092.

MOURIDSEN, H.T., PALSHOF, T., BRAHM, M. & 4 others (1976). Evaluation of single-drug vs. multiple-drug chemotherapy in metastatic carcinoma of the breast. *Cancer Res.*, **36**, 3911.

NEMOTO, T., ROSNER, D., DIAZ, R. & 4 others (1978). Combination chemotherapy for metastatic breast cancer. *Cancer*, **41**, 2073.

PETO, R., PIKE, M.C., ARMITAGE, P. & 8 others (1977). Design and analysis of randomised clinical trials requiring prolonged observation of each patient. *Br. J. Cancer*, **35**, 1.

RUBENS, R.D., KNIGHT, R. & HAYWARD, J.L. (1975). Chemotherapy of advanced breast cancer: a controlled randomized trial of cyclophosphamide versus a four-drug combination. *Br. J. Cancer.*, **32**, 730.

SACKS, H.S., BERRIER, J., REITMAN, D. & 3 others (1987). Meta-analyses of randomized controlled trials. *N. Engl. J. Med.*, **316**, 450.

SMALLEY, R.V., MURPHY, R. & HUGULEY, J.L. (1976). Combination versus sequential five-drug chemotherapy in metastatic carcinoma of the breast. *Cancer Res.*, **36**, 3911.

TAYLOR, S.G., GELMAN, R.S., FALKSON, G. & 4 others (1986). Combination chemotherapy compared to tamoxifen as initial therapy for Stage IV breast cancer in elderly women. *Ann. Intern. Med.*, **104**, 455.

ZINSER, J.W., HORTOBAGYI, G.N., BUZDAR, A.U. & 4 others (1987). Clinical course of breast cancer patients with liver metastases. *J. Clin. Oncol.*, **5**, 773.


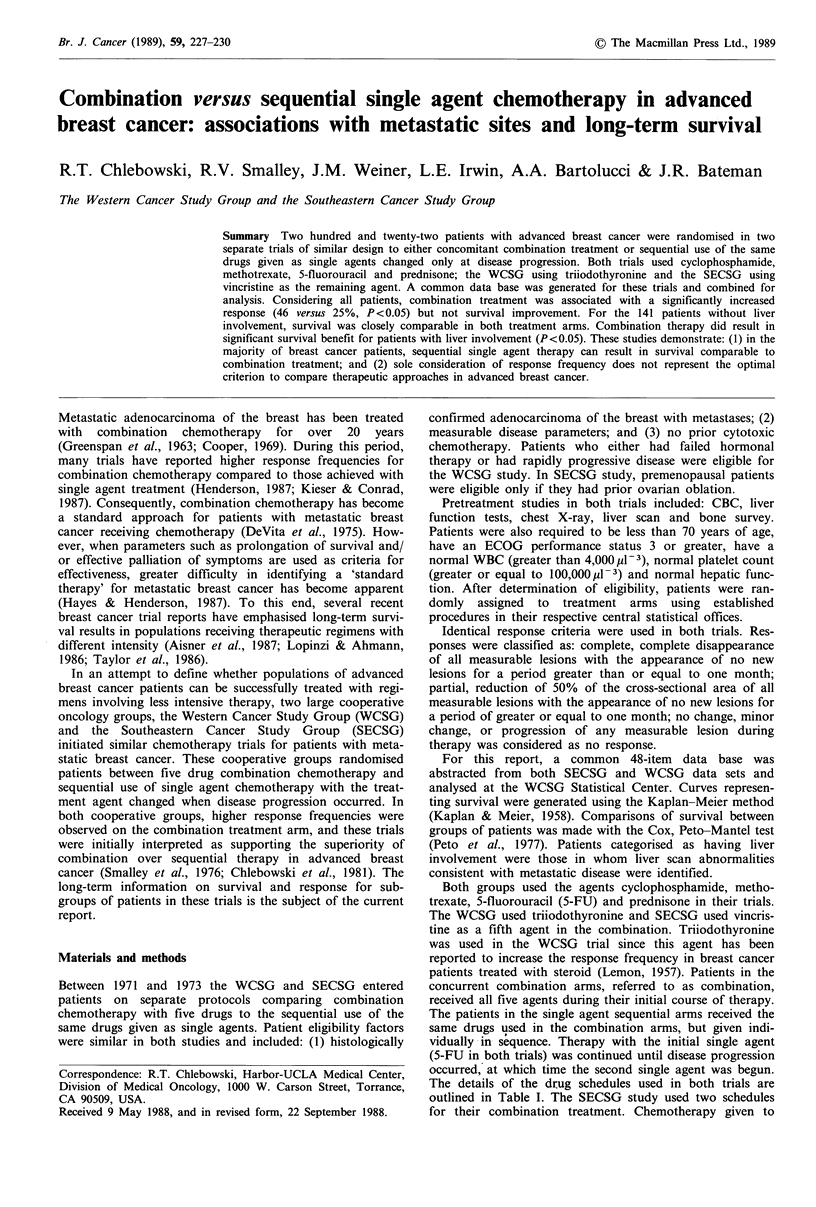

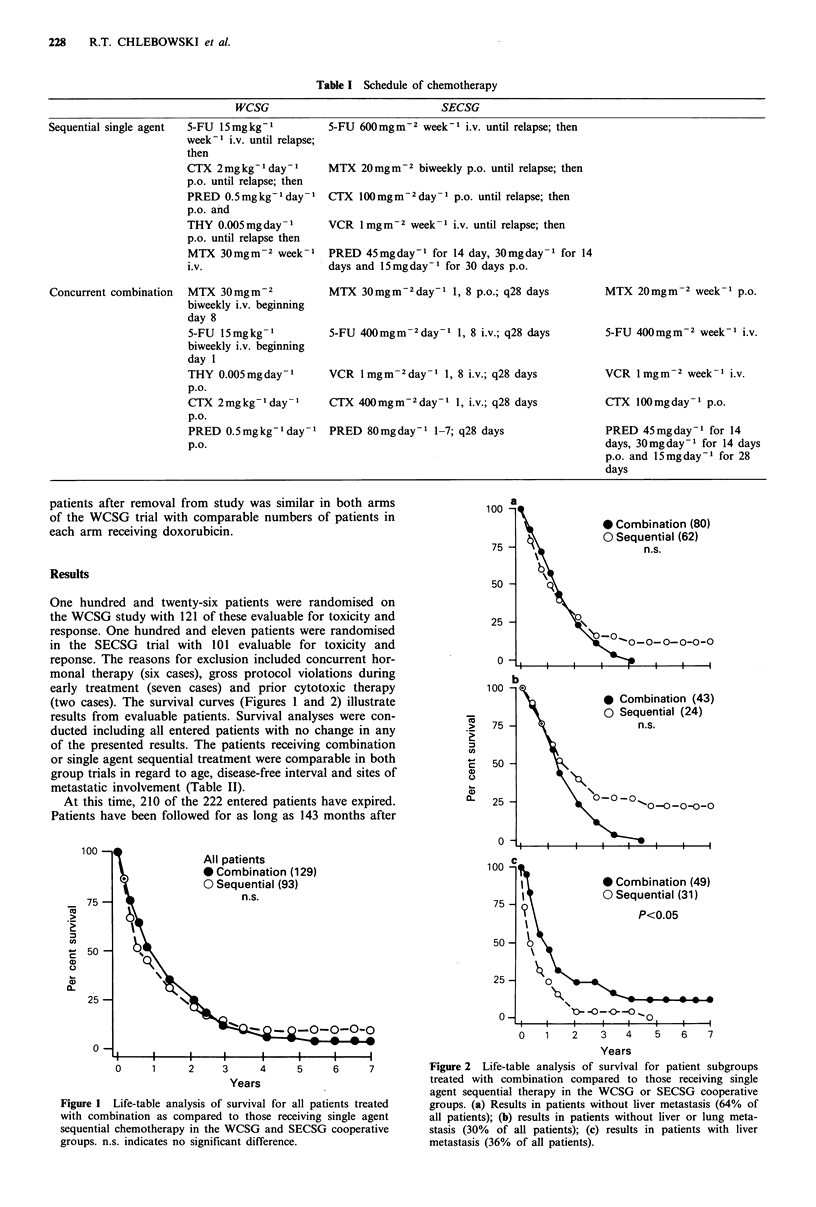

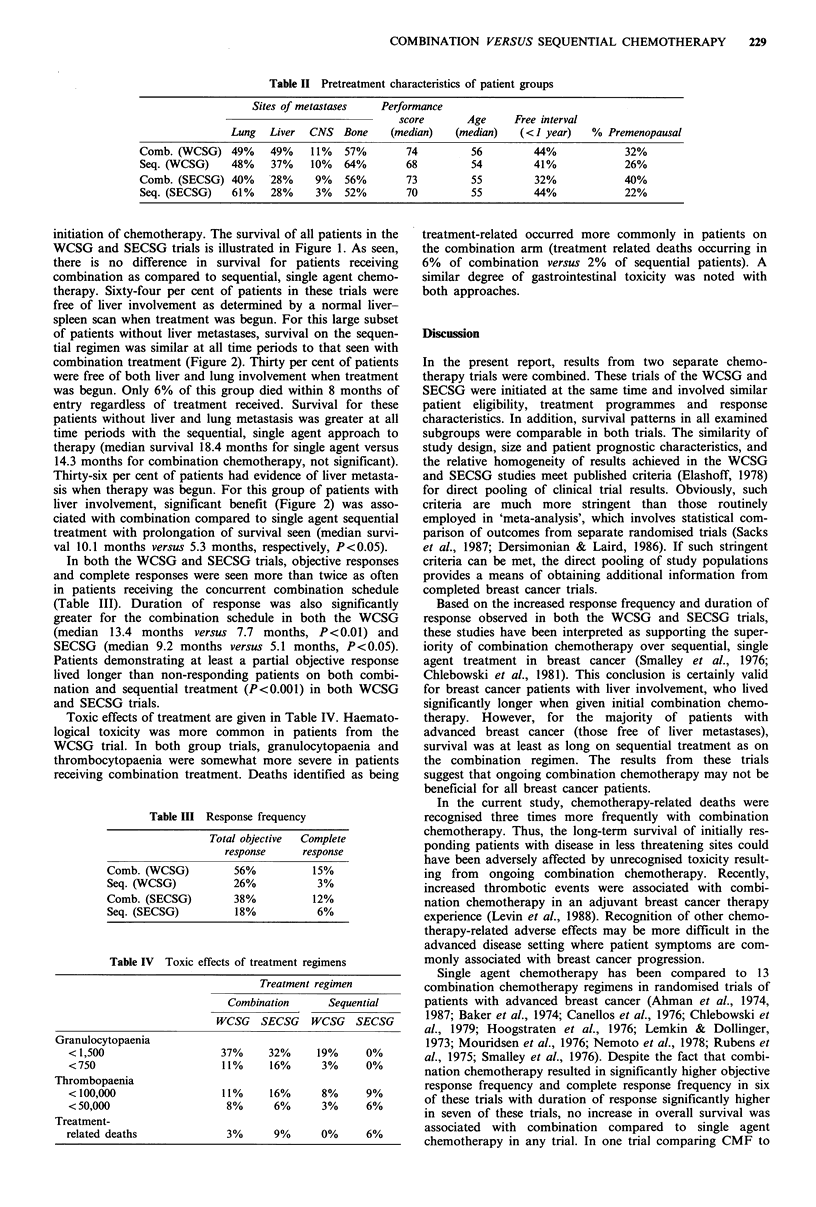

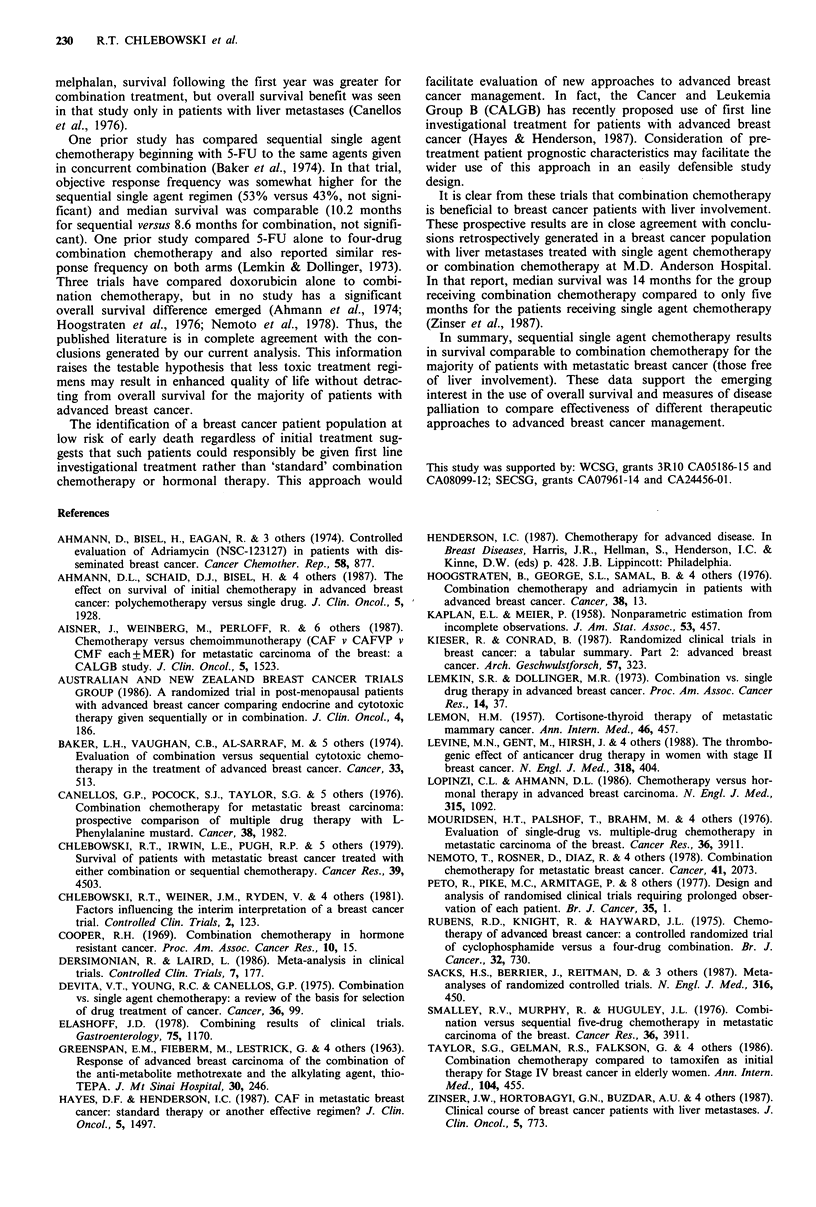


## References

[OCR_00553] Ahmann D. L., Bisel H. F., Eagan R. T., Edmonson J. H., Hahn R. G. (1974). Controlled evaluation of adriamycin (NSC-123127) in patients with disseminated breast cancer.. Cancer Chemother Rep.

[OCR_00558] Ahmann D. L., Schaid D. J., Bisel H. F., Hahn R. G., Edmonson J. H., Ingle J. N. (1987). The effect on survival of initial chemotherapy in advanced breast cancer: polychemotherapy versus single drug.. J Clin Oncol.

[OCR_00564] Aisner J., Weinberg V., Perloff M., Weiss R., Perry M., Korzun A., Ginsberg S., Holland J. F. (1987). Chemotherapy versus chemoimmunotherapy (CAF v CAFVP v CMF each +/- MER) for metastatic carcinoma of the breast: a CALGB study. Cancer and Leukemia Group B.. J Clin Oncol.

[OCR_00577] Baker L. H., Vaughn C. B., al-Sarraf M., Reed M. L., Vaitkevicius V. K. (1974). Proceedings: Evaluation of combination vs. sequential cytotoxic chemotherapy in the treatment of advanced breast cancer.. Cancer.

[OCR_00661] (1986). Chemotherapy versus hormonal therapy in advanced breast carcinoma.. N Engl J Med.

[OCR_00589] Chlebowski R. T., Irwin L. E., Pugh R. P., Sadoff L., Hestorff R., Wiener J. M., Bateman J. R. (1979). Survival of patients with metastatic breast cancer treated with either combination or sequential chemotherapy.. Cancer Res.

[OCR_00595] Chlebowski R. T., Weiner J. M., Ryden V. M., Bateman J. R. (1981). Factors influencing the interim interpretation of a breast cancer trial: danger of achieving the "expected" result.. Control Clin Trials.

[OCR_00604] DerSimonian R., Laird N. (1986). Meta-analysis in clinical trials.. Control Clin Trials.

[OCR_00613] Elashoff J. D. (1978). Combining results of clinical trials.. Gastroenterology.

[OCR_00617] GREENSPAN E. M., FIEBER M., LESNICK G., EDELMAN S. (1963). Response of advanced breast carcinoma to the combination of the antimetabolite, Methotrexate, and the alkylating agent, thio-TEPA.. J Mt Sinai Hosp N Y.

[OCR_00623] Hayes D. F., Henderson I. C. (1987). CAF in metastatic breast cancer: standard therapy or another effective regimen?. J Clin Oncol.

[OCR_00642] Kieser R., Conrad B. (1987). Randomized clinical trials in breast cancer: a tabular summary. Part 2: Advanced breast cancer.. Arch Geschwulstforsch.

[OCR_00652] LEMON H. M. (1957). Cortisone-thyroid therapy of metastatic mammary cancer.. Ann Intern Med.

[OCR_00656] Levine M. N., Gent M., Hirsh J., Arnold A., Goodyear M. D., Hryniuk W., De Pauw S. (1988). The thrombogenic effect of anticancer drug therapy in women with stage II breast cancer.. N Engl J Med.

[OCR_00671] Nemoto T., Rosner D., Diaz R., Dao T., Sponzo R., Cunningham T., Horton J., Simon R. (1978). Combination chemotherapy for metastatic breast cancer: comparison of multiple drug therapy with 5-fluorouracil, cytoxan and prednisone with adriamycin or adrenalectomy.. Cancer.

[OCR_00680] Rubens R. D., Knight R. K., Hayward J. L. (1975). Chemotherapy of advanced breast cancer: a controlled randomized trial of cyclophosphamide versus a four-drug combination.. Br J Cancer.

[OCR_00686] Sacks H. S., Berrier J., Reitman D., Ancona-Berk V. A., Chalmers T. C. (1987). Meta-analyses of randomized controlled trials.. N Engl J Med.

[OCR_00691] Smalley R. V., Murphy S., Huguley C. M., Bartolucci A. A. (1976). Combination versus sequential five-drug chemotherapy in metastatic carcinoma of the breast.. Cancer Res.

[OCR_00696] Taylor S. G., Gelman R. S., Falkson G., Cummings F. J. (1986). Combination chemotherapy compared to tamoxifen as initial therapy for stage IV breast cancer in elderly women.. Ann Intern Med.

[OCR_00702] Zinser J. W., Hortobagyi G. N., Buzdar A. U., Smith T. L., Fraschini G. (1987). Clinical course of breast cancer patients with liver metastases.. J Clin Oncol.

